# Evolution Mechanisms of Microstructure and Performance of Aluminum Alloy Thin-Walled Components Repaired by Friction Stir Spot Welding

**DOI:** 10.3390/ma19173567

**Published:** 2026-08-22

**Authors:** Xiaoming Ye, Jie Zhang, Yuan Liu, Qiu Pang, Yuwei Li

**Affiliations:** 1 State Key Laboratory of Light Superalloys, Wuhan University of Technology, Wuhan 430070, China; yexm@dfmc.com.cn (X.Y.); lixiaotuans@163.com (Y.L.); 2 Research Institute of Dongfeng Motor Group Co., Ltd., Wuhan 430058, China; 3 Department of Mechanical and Electrical Engineering, Wuhan Donghu University, Wuhan 430212, China; zhangjie@wdu.edu.cn (J.Z.); liuyuan@wdu.edu.cn (Y.L.)

**Keywords:** aluminum alloy thin-walled parts, numerical simulation, material flow, mechanical property

## Abstract

Taking the repair of prefabricated hole defects in 2024 aluminum alloy thin-walled components by friction stir spot welding (FSSW) as the research object, the evolution laws of microstructure and mechanical properties of FSSW-repaired joints of thin-walled components were clarified through process experiments and numerical simulations. The collaborative effect of the temperature field and material flow field during the FSSW repair process and their regulation laws on the microstructure and properties were revealed. The results show that as the repair speed increases, the macroscopic surface quality of the FSSW joint improves. When the repair speed reaches 2000 r/min, a high-quality repaired joint with a smooth and flat surface and no porosity defects can be obtained. Meanwhile, within the repair speed range of 800 to 2000 r/min, the grains undergo dynamic recrystallization (DRX) due to the combined effect of heat and mechanical forces, eventually forming a uniform equiaxed grain structure in the weld core area. ABAQUS 2023 simulation verifies the temperature distribution during the FSSW repair process. When the repair speed is 2000 r/min, the maximum temperature obtained from the simulation is 431.1 °C, which agrees with the measured value from the experiment. The simulation results further reveal that when the repair speed increases from 1200 r/min to 2000 r/min, the material fluidity significantly enhances, and the flow velocity on the advancing side is always higher than that in other areas. At the rotational speed of 2000 r/min, the plastic material flows continuously from the periphery and eventually fills the defect area completely. The fracture mode of the FSSW-repaired joint is mainly ductile fracture. With the increase in the repair speed, the number of dimples at the fracture surface increases significantly. When the rotational speed reaches 2000 r/min, the joint achieves the best mechanical properties, and the FSSW-repaired joint reaches the maximum tensile strength of 169 MPa.

## 1. Introduction

In the aerospace industry, the demand for reducing the weight of components to enhance fuel efficiency is increasing. Lightweighting has become an eternal theme in the research and development of aerospace equipment [1,2,3]. Especially, 2xxx and 7xxx series aluminum alloys, with their low density, high specific strength, good toughness, and excellent corrosion resistance, are widely used in the aerospace industry. However, perforation and hole defects are common forms of damage in key load-bearing structures of aviation, mainly occurring in aircraft skins, engines, fan blades, and other aviation key components under harsh working conditions. Due to the high value, high performance, harsh working conditions, short service life, and the low remanufacturing rate of such critical components, the manufacturing of aerospace equipment causes significant resource waste [4,5,6]. Therefore, repairing and remanufacturing defects in aluminum alloy components is an effective means to achieve cost reduction and efficiency improvement [7].

At present, the main methods for repairing hole defects in aluminum alloys include traditional fusion welding, laser cladding, electrical discharge alloying, and friction stir welding. However, traditional fusion welding techniques often result in coarse grains due to the high local temperature. Laser cladding and electrical discharge alloying are only suitable for repairing surface defects, and it is rather difficult to repair internal defects in structural components with these methods [8,9]. In addition, the large amount of heat generated during the welding process can cause severe deformation of the components. Friction stir welding can repair small volume defects such as surface cracks, grooves, and tunnels on aluminum alloys, but the repair process for hole-like defects and large-area, large-volume surface defects is more complex and difficult to achieve effective repair.

Friction stir spot welding (FSSW) is a novel solid-state joining technology suitable for aluminum alloys. It generates heat through the friction between the shoulder of the stirring tool and the workpiece surface, causing the metal at the weld point to undergo plastic flow and form a metallurgical bond without keyhole defects. Compared with traditional methods, it has advantages such as low welding temperature, small residual stress, high joint quality, and low production cost. It can better adapt to the characteristics of aluminum alloys and has broad application prospects in the fields of aviation, aerospace, and automotive manufacturing [10,11,12]. In recent years, domestic and international scholars have initially explored and attempted to utilize the FSSW technology to repair defects in materials of different forms, including backfill friction stir spot welding repair, filler friction stir welding repair, and friction plug welding repair. By optimizing the welding repair parameters, the flow of weld metal in the welded materials is promoted, effectively repairing the hole defects and forming uniform and dense repaired joints, thereby improving the repair efficiency and quality [13,14].

Reimann et al. [15] conducted friction stir spot welding hole repair on 6 mm thick AA 7075-T651 plates and investigated the evolution of thermal cycles and microstructure characteristics. The results showed that the heating rate at the weld center was high, with the peak temperature reaching up to 540 °C, and the minimum hardness was 70% of the base material hardness. Liu LA et al. [16] employed filling friction stir welding to perform a quasi-equal-strength solid-state repair on the keyhole defect in a 6 mm-thick 2195 aluminum–lithium alloy weld. The results indicated that under the rotational speed of 1600 r/min, the repaired joint achieved 90.7% of the high-quality friction stir welding joint, and its tensile strength reached 358.1 MPa. Xiong [17] et al. used the ABAQUS 2020 software to investigate the influence of different rotational speeds on the filling of voids in A2524 aluminum alloy. The results showed that with the rotation speed increasing from 2200 rpm to 2800 rpm, the flow of plastic metal improved, leading to a significant decrease in voids. However, current research by domestic and international scholars on friction stir spot welding (FSSW) repair techniques is mainly focused on low rotational speed parameters [18]. Most studies suggest that lower rotational speeds help reduce heat input during the repair process, thereby effectively suppressing softening in the heat-affected zone of the repaired area, avoiding excessive material deformation or defects such as flash, and facilitating the formation of repaired joints with uniform microstructure and good mechanical properties. However, research on material flow behavior, interfacial bonding mechanisms, and microstructure evolution of the repaired layer under high rotational speed conditions during FSSW repair remains relatively limited, and the relevant mechanisms require further in-depth investigation.

This paper takes prefabricated hole defects in 2024 aluminum alloy thin-walled components as the research object and proposes a friction stir spot welding (FSSW) repair process for such components. By combining process experiments with numerical simulation, the evolution laws of temperature field, material flow, microstructure, and mechanical properties of 2024 aluminum alloy thin-walled component joints under different repair process parameters are investigated. The numerical simulation of temperature distribution and material flow filling behavior in the FSSW-repaired joints of thin-walled components is revealed, and FSSW-repaired joints of aluminum alloy thin-walled components with good repair performance are obtained, providing a scientific basis for practical engineering applications.

## 2. Materials and Methods

**Figure 1:**
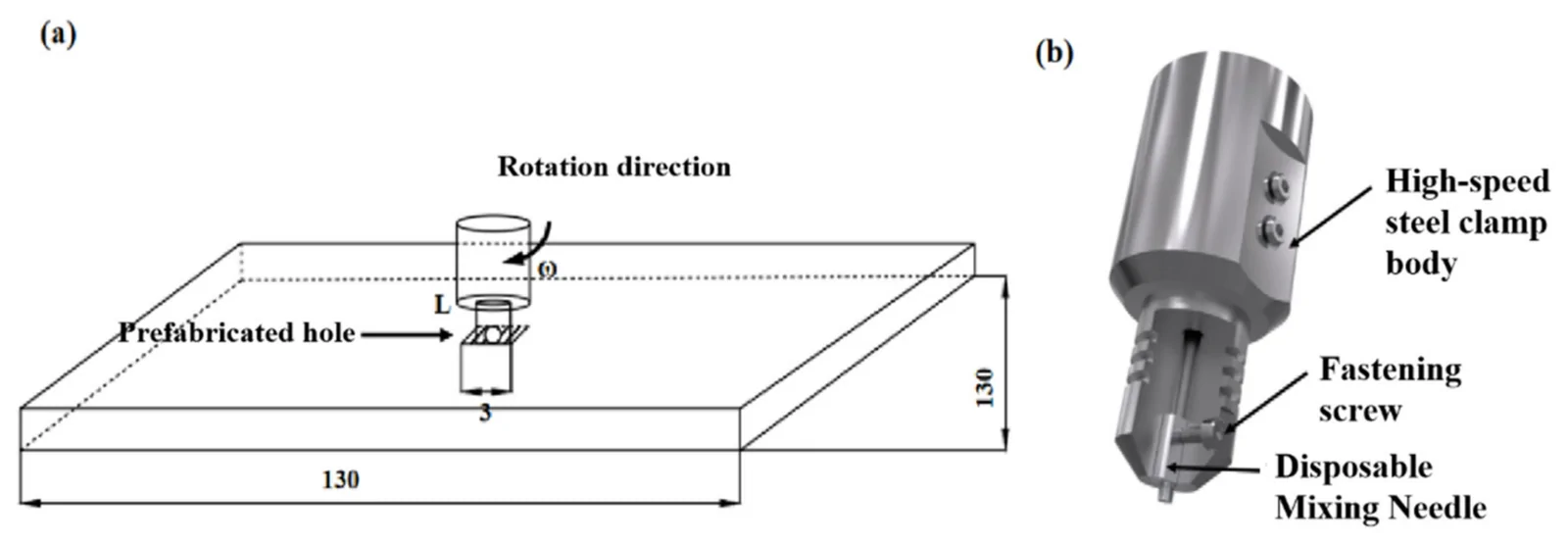
Schematic diagram of the FSSW repair process and the morphology of the repair mixing head. (**a**) Schematic diagram of the FSSW repair process; (**b**) the morphology of the mixing head.

The repair material used in the FSSW repair process is a 3 mm thick 2024-O aluminum alloy sheet, which was purchased from Nanna Aluminum Co., Ltd. (Nanning, China). The chemical composition and mechanical properties of 2024-O aluminum alloy are shown in Table 1. The dimensions are 330 mm in length, 130 mm in width, and 3 mm in thickness, and there is a pre-made hole defect in the middle with a size of Φ 3 mm × 1.5 mm. Figure 1 shows the NFSW-650 model FSSW repair process equipment developed by the Chinese Academy of Sciences Institute. The welding speed and rotational speed of the stirring head can be adjusted according to different welding repair requirements and material properties to achieve the best welding effect. During the FSSW repair process of thin-walled components, a testo 890 infrared thermal imager was used to acquire the instantaneous temperatures in the region near the stirring tool under different welding process parameters.

Figure 1 presents the illustration of the FSSW repair process for thin-walled components and the morphology of the stirring head. The FSSW repair process, as illustrated in Figure 1a, comprises three stages: (1) Positioning: The spindle aligns its axis with the center of the pre-existing hole in the workpiece. (2) Plunging and Filling: The rotating spindle drives the consumable tool, plunging it into the hole defect. The material is plasticized and displaced, completely filling the cavity. (3) Retraction: Upon fracture of the consumable pin, the spindle ceases rotation and retracts to its initial position. In this paper, the FSSW repair parameters for thin-walled components are set as follows: The FSSW process is carried out in the vertical direction, with the tool descending at a constant speed of 20 mm/min. The rotational speeds of 800, 1200, 1600, and 2000 rpm are used. The welding process is position-controlled. The dwell time applied in this study is 5 s for the tool within the defect hole. The plunge depth of the tool shoulder reaches 0.1 mm.

Figure 1b shows the morphology of the FSSW repair tool for thin-walled components. Based on this repair process, a split-type tool is independently designed, which consists of a high-speed steel holder, a consumable stirring pin, and a fastening screw. The material of the clamping body is H13 steel, and the stirring pin is made of 2024 aluminum alloy of the same material as the base material. The upper part of the stirring pin is designed in a flat shape to connect with the clamping body and is fixed by a fastening screw on the outside. The lower part is designed in a long conical shape to consume the filled hole defects. To achieve a better filling effect, the stirring tool is a cone with a right-hand thread, with a shoulder diameter of 3 mm and a pin length of 3.05 mm.

**Figure 2:**
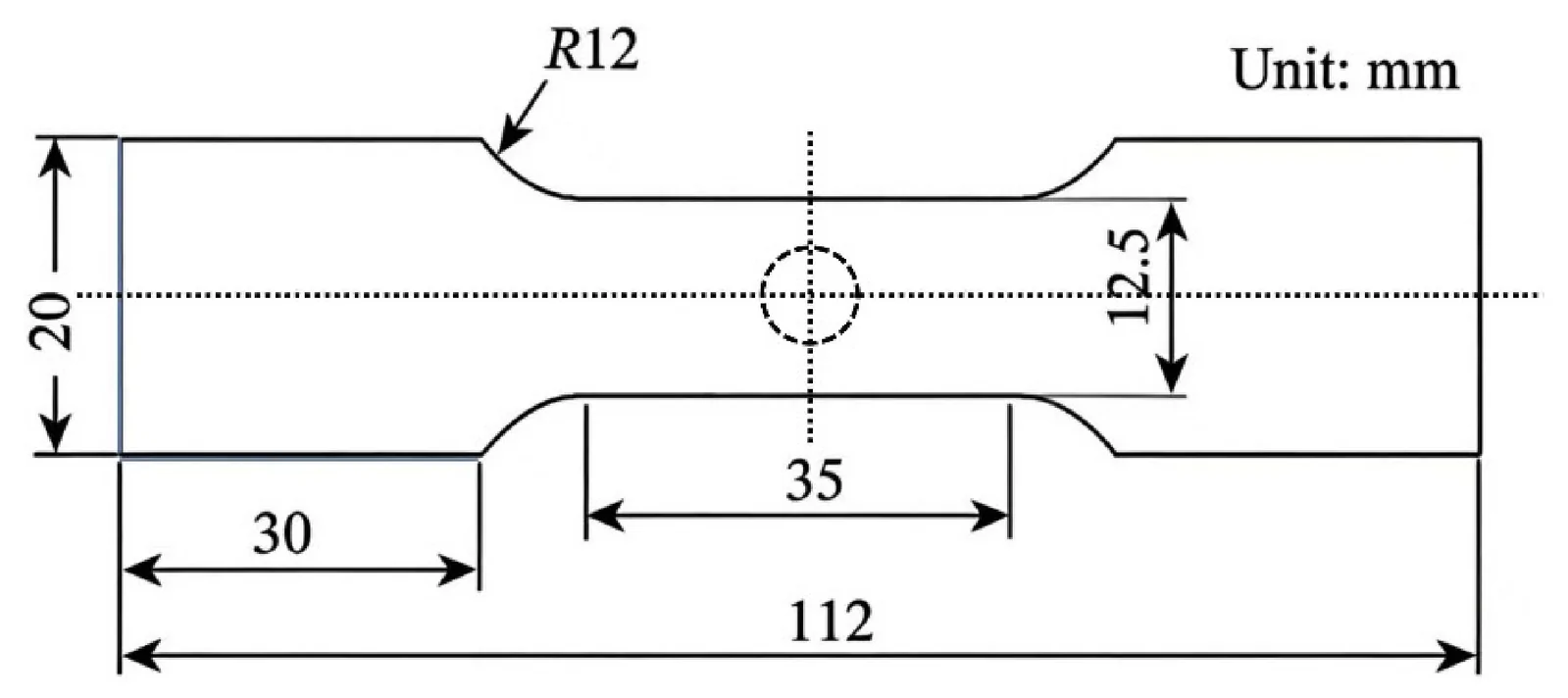
Tensile specimen size of FSSW-repaired joint at room temperature.

A 15 mm × 3 mm × 3 mm sample block is cut along the central axis of the repair area using a wire-cutting machine to prepare a metallographic specimen, and then it is successively ground with different grades of sandpaper. After polishing, the metallographic samples are etched with Keller’s reagent (1 mL HF + 1.5 mL HCl + 2.5 mL HNO_3_ + 95 mL H_2_O). The macroscopic and microscopic morphologies of the cross-section of the FSSW-repaired joint are observed using a Zeiss Scope Al metallographic microscope (Carl Zeiss Microscopy GmbH, Jena, Germany) and a Zeiss Ultra Plus scanning electron microscope (Carl Zeiss Microscopy GmbH, Oberkochen, Germany). The hardness test of the FSSW-repaired joint is conducted using an HV-1000 microhardness tester, with a loading force of 200 g and a dwell time of 15 s. According to GB/T228.1-2021 “Metallic Materials—Tensile Testing—Part 1: Method of Test at Room Temperature” [19], the centerline of the tensile specimen is perpendicular to the spot welding direction. The schematic diagram of the tensile specimen for the FSSW-repaired joint is shown in Figure 2. Unidirectional tensile tests at room temperature are conducted on FSSW-repaired joints with different repair process parameters using a WDW-100 electronic universal testing machine at a rate of 2 mm/min. Each process parameter is tested three times and the average value is taken.

**Figure 3:**
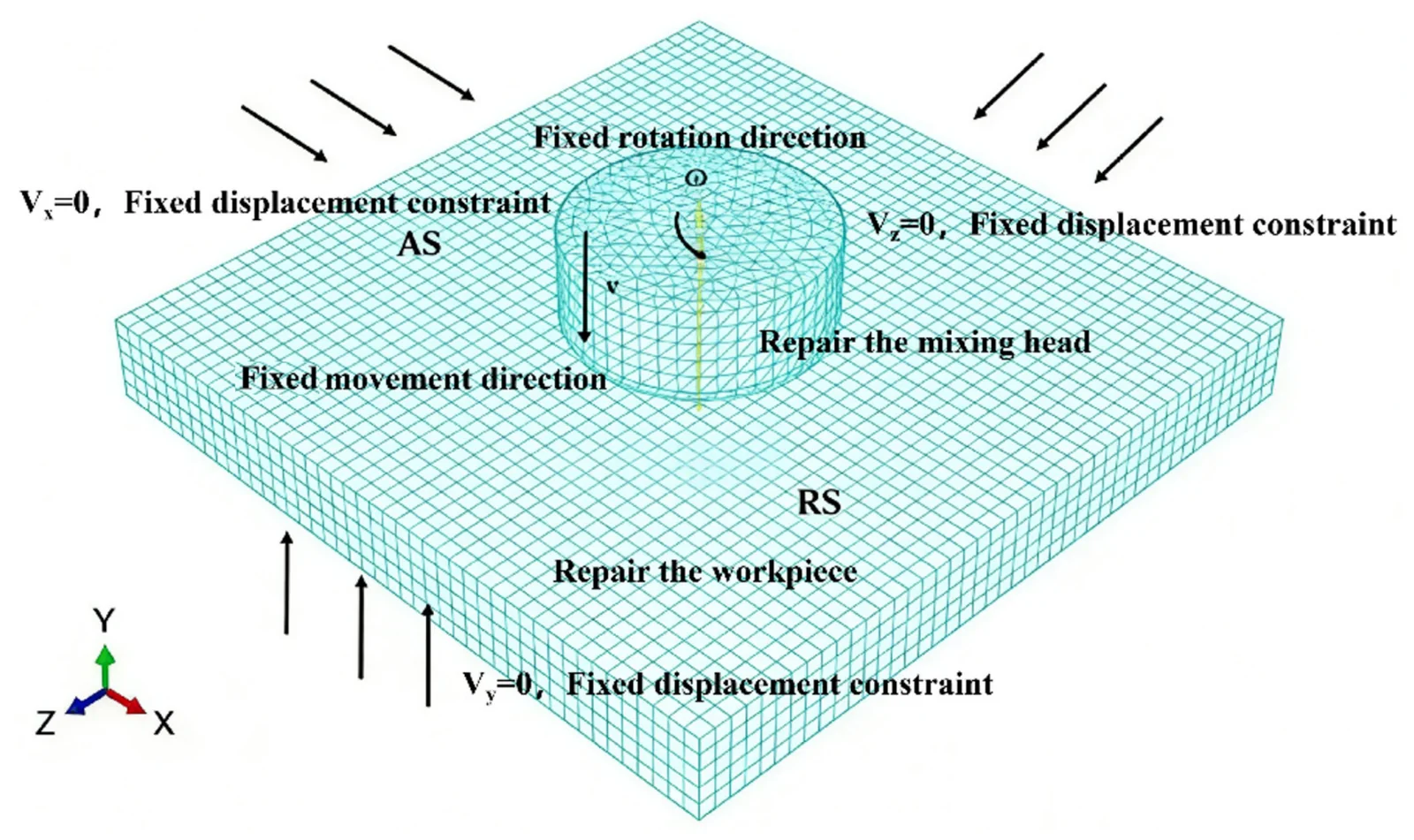
Thermodynamic coupling model of FSSW repair.

**Figure 4:**
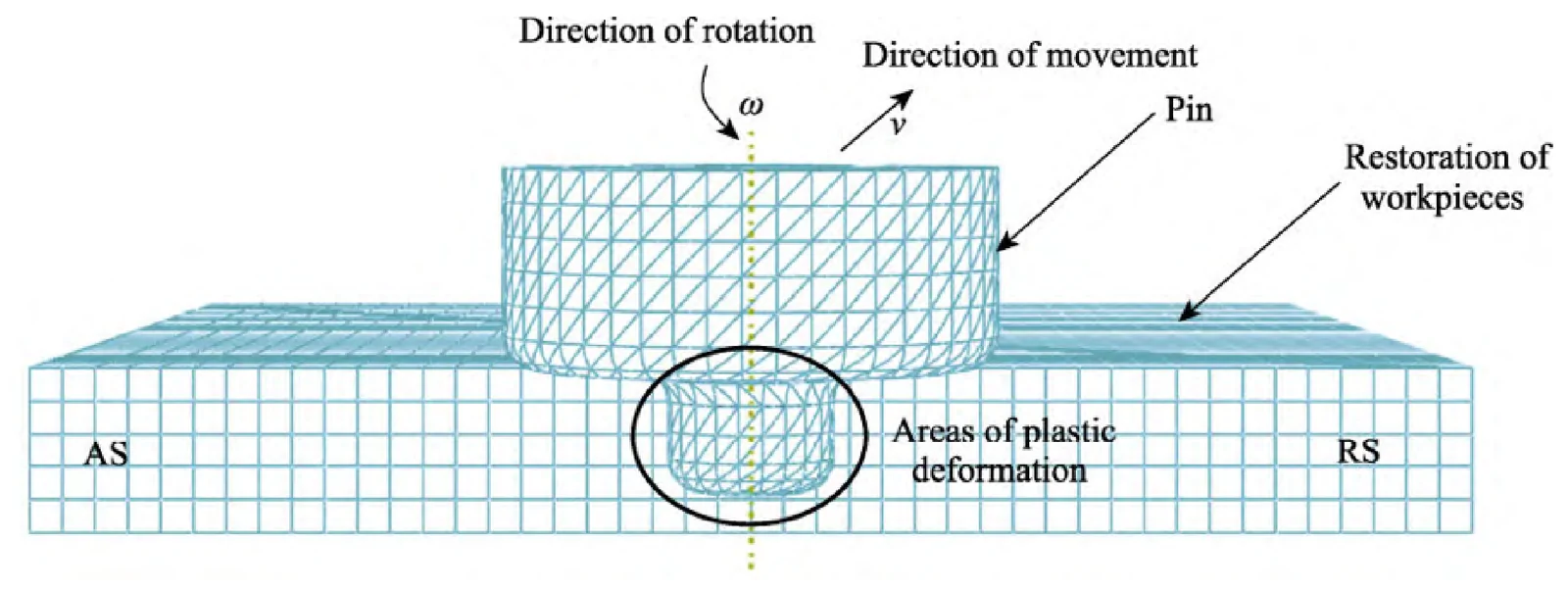
The cross-sectional view of thermodynamic coupling model of FSSW repair.

A thermal–mechanical coupled model for FSSW repair is established using the ABAQUS finite element software, and the stirring head is set as a rigid body, as shown in Figure 3 and Figure 4. The vertical force is 10 kN, and the plunging/lifting speed is 20 mm/min. The slip rate is taken as 0.5, and the friction coefficient (μ) is set to 0.3. To ensure calculation accuracy and reduce solution time, the mesh around the stirring head within a 0.4 mm radius was refined. The specific mesh type was a tetrahedral 10-node temperature-displacement coupled element, C3D10MT. For the mesh far from the stirring head, the type was a hexahedral 8-node coupled element, EC3D8RT. Meanwhile, the boundary conditions between the stirring head and the repaired workpiece were set as general contact constraints. The repair rotational speeds of the stirring head were the same as that of the test, with speeds of 800 r/min, 1200 r/min, 1600 r/min, and 2000 r/min applied. The speeds of V_X_, V_Y_, and V_Z_ were all 0 and were fixed displacement constraints. Finally, the explicit dynamics analysis was adopted for the solution.

In the FSSW repair thermal coupling model, FSSW repair is a complex coupled thermodynamic problem in which heat is mainly generated by the friction between the repair stir head and the repair workpiece as well as plastic deformation. In this model, the plastic deformation heat $Q_{p}$ was generated by the non-elastic work within the shear layer of the repaired workpiece under adhesive conditions, as shown in Equation (1):

(1) $$Q_{p}=\eta \sigma_{fs}\varepsilon_{pl}$$

where η represents the thermal conversion efficiency within the repaired workpiece. $\varepsilon_{pl}$ is the strain rate. $\sigma_{fs}$ is the flow stress of the repair material.

During the FSSW repair process, the frictional heat $Q_{f}$ generated by the sliding friction between the stirring head and the repaired workpiece is expressed as shown in Formula (2):

(2) $$Q_{p}=\tau \gamma$$

where γ represents the slip rate. τ is the frictional shear stress.

According to Coulomb’s law of friction, τ can be expressed as:

***τ* = *μ p*** (3)

where p is the contact pressure at the interface between the stirring head and the repaired workpiece. *μ* is the friction coefficient, with a value of 0.3.

During the FSSW repair process, considering that some heat is lost from the workpiece surface through convection and radiation, the corresponding heat transfer boundary conditions are as shown in Formula (4):

(4) $$-K\frac{\partial T}{\partial n_{s}}=\sigma_{b}\varepsilon_{b}\left(T^{4}-T_{a}^{4}\right)+h_{con}\left(T-T_{a}\right)$$

where $n_{s}$ is the distance between isothermal surfaces in the normal direction of the re paired workpiece. $\sigma_{b}$ is the Stefan–Boltzmann constant with a value of 5.67 × 10^−8^ W/m^2^·K^4^. $\varepsilon_{b}$ is the emissive value of 0.11. $T_{a}$ is the ambient temperature. $h_{con}$ is the convective heat transfer coefficient with a value of 10.2 W/m^2^ °C.

The Johnson–Cook material law is adopted to describe the plasticity of the workpiece material. The Johnson–Cook material law parameters are listed in Table 2. The flow stress is defined as follows:

(5) $$\sigma_{fs}=\left(A+B\varepsilon_{pl}^{n}\right)\left(1+Cln\frac{\varepsilon_{pl}}{\varepsilon_{0}}\right)\left(1-{\left(\frac{T-T_{ref}}{T_{melt}-T_{ref}}\right)}^{m}\right)$$

where $\varepsilon_{pl}$ is the strain rate after repair. $\varepsilon_{p}$ is the equivalent plastic strain rate. **ε_0_** is the strain rate before repair, and A, B, C, n, and m are material constants. The exponent n represents the strain hardening effect, m is the thermal softening exponent, and C is the strain rate strengthening parameter. The parameters **A**, **B** and **n** are experimentally evaluated at temperature **T_ref_**, and **T_melt_** is the solidus temperature of the material.

## 3. Results and Discussion

**Figure 5:**
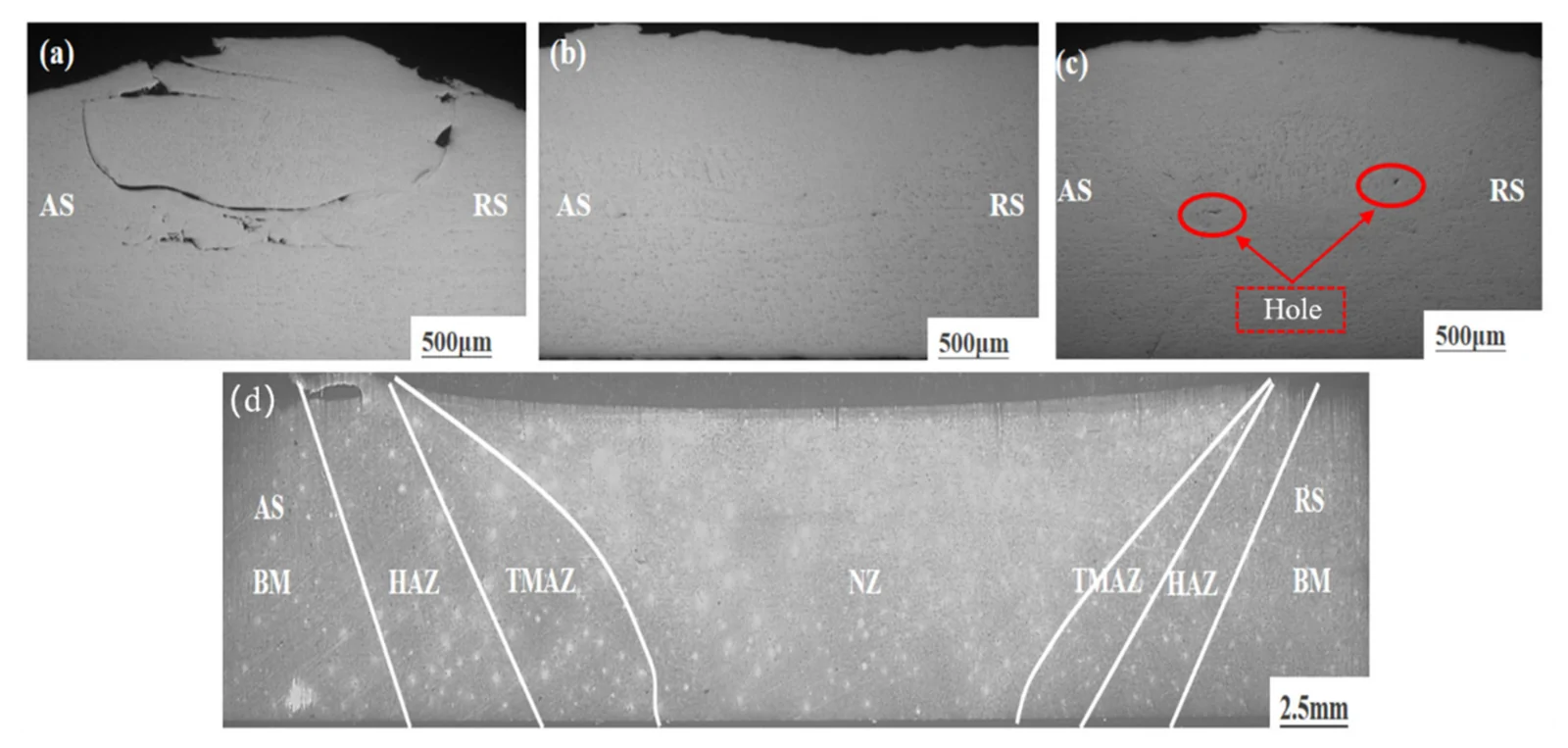
Cross-sectional morphology of the FSSW-repaired joint under different repair speeds: (**a**) 800 r/min; (**b**) 1200 r/min; (**c**) 1600 r/min; and (**d**) 2000 r/min.

Figure 5 shows the cross-sectional morphology of the FSSW-repaired joints of thin-walled components with a repair welding speed of 20 mm/min and repair rotational speeds of 800 r/min, 1200 r/min, 1600 r/min, and 2000 r/min. In the figure, AS and RS represent the advancing side and retreating side of the repaired joint, respectively. Under the same repair welding speed, the FSSW-repaired joints of 2024 aluminum alloy with hole defects present different microstructures as the repair rotational speed increases. When the rotational speed is 800 r/min, a wide range of pores are generated around the FSSW repair stirring head. The material of the stirring head does not fully fuse with the base material, and there are large holes in some areas, as shown in Figure 5a. When the rotational speed is 1200 r/min, there are no significant repair defects in the cross-section of the FSSW-repaired joint, but there are tiny holes at the bottom of the connection of the stirring head. When the repair speed is increased to 1600 r/min, the microstructure morphology of the cross-section of the FSSW-repaired joint is relatively smooth, but there are some tiny hole defects in the cross-section, as shown in Figure 5c. When the rotational speed reaches 2000 r/min, the material undergoes sufficient plastic deformation, and no interface between the weld core material and the base material appears around the weld core. The cross-section of the repaired joint shows a symmetrical feature [20], as shown in Figure 5d. The microstructure characterization of the FSSW joint shows that it can be divided into four typical regions from the repair center to the edge: the Nugget Zone (NZ), the thermomechanical affected zone (TMAZ), the heat affected zone (HAZ), and the base metal zone (BM) [21]. Further observation reveals that the bottom of the repair zone achieves a complete metallurgical bond with the BM, and at the same time, the microstructure in the central NZ zone is uniform without any void defects.

**Figure 6:**
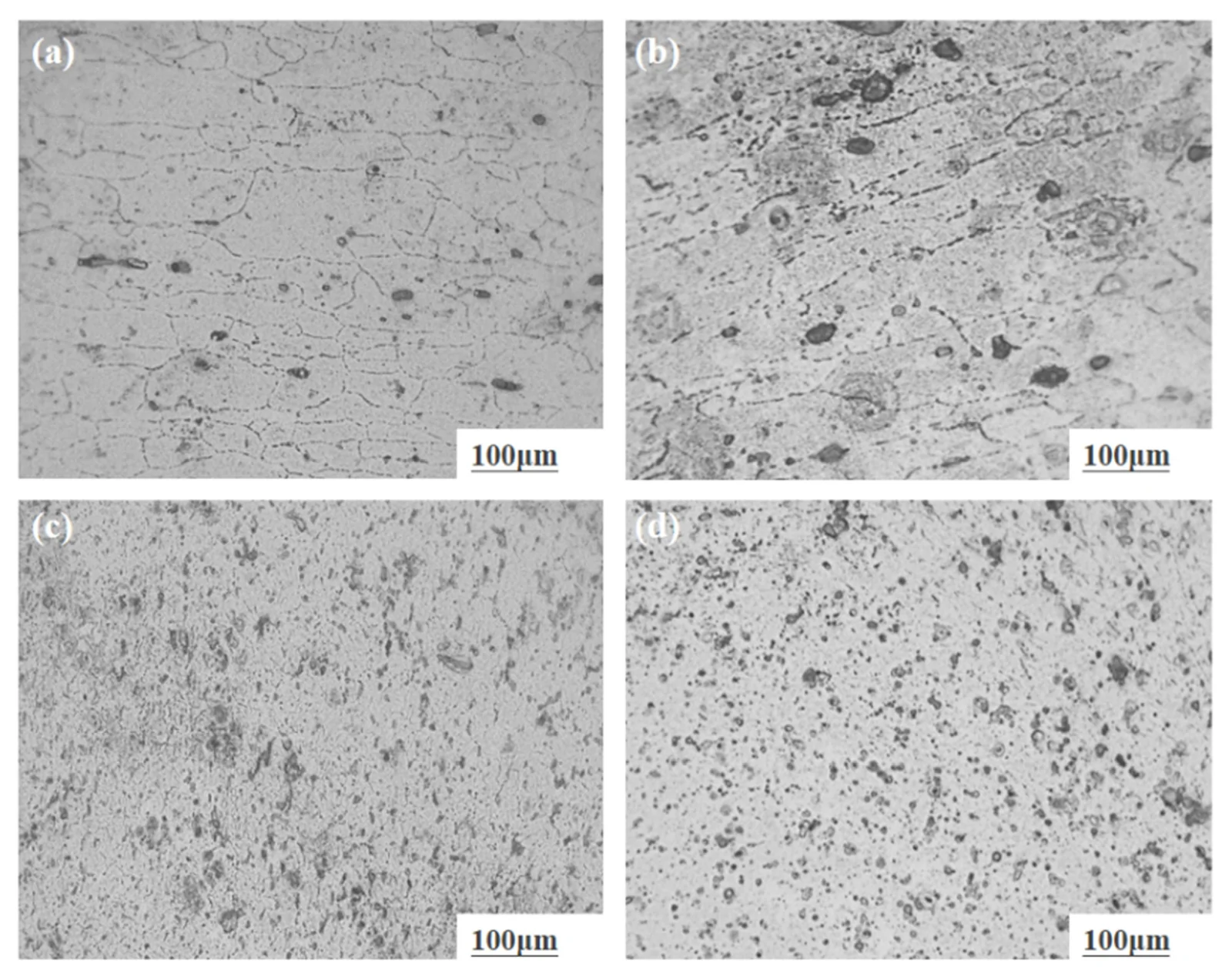
Microstructure morphologies of various zones of the FSSW-repaired joint at a repair rotation speed of 1600 r/min: (**a**) BM zone; (**b**) HAZ zone; (**c**) TMAZ zone; and (**d**) NZ zone.

Figure 6 shows the microstructure morphologies of various zones in the repaired joint under the conditions of a repair rotation speed of 1600 r/min. In the BM and HAZ zones, due to the greater distance from the stirring tool and thus lower heat input, the grains in the BM zone of the FSSW-repaired joint are relatively large and exhibit an elongated distribution, while the grain size in the HAZ zone shows essentially no obvious change compared with the base material, as shown in Figure 6a,b. In the TMAZ zone, the heat input is significantly higher than that in the HAZ zone, and during the process of filling the repaired hole, the grains are refined under the intense mechanical stirring action of the tool, as shown in Figure 6c. In the NZ zone, under the combined influence of substantial mechanical stirring and heat input from the tool, the grains undergo significant bending and plastic deformation. The closer to the edge of the stirring tool, the more pronounced this grain changes become, as shown in Figure 6d.

**Figure 7:**
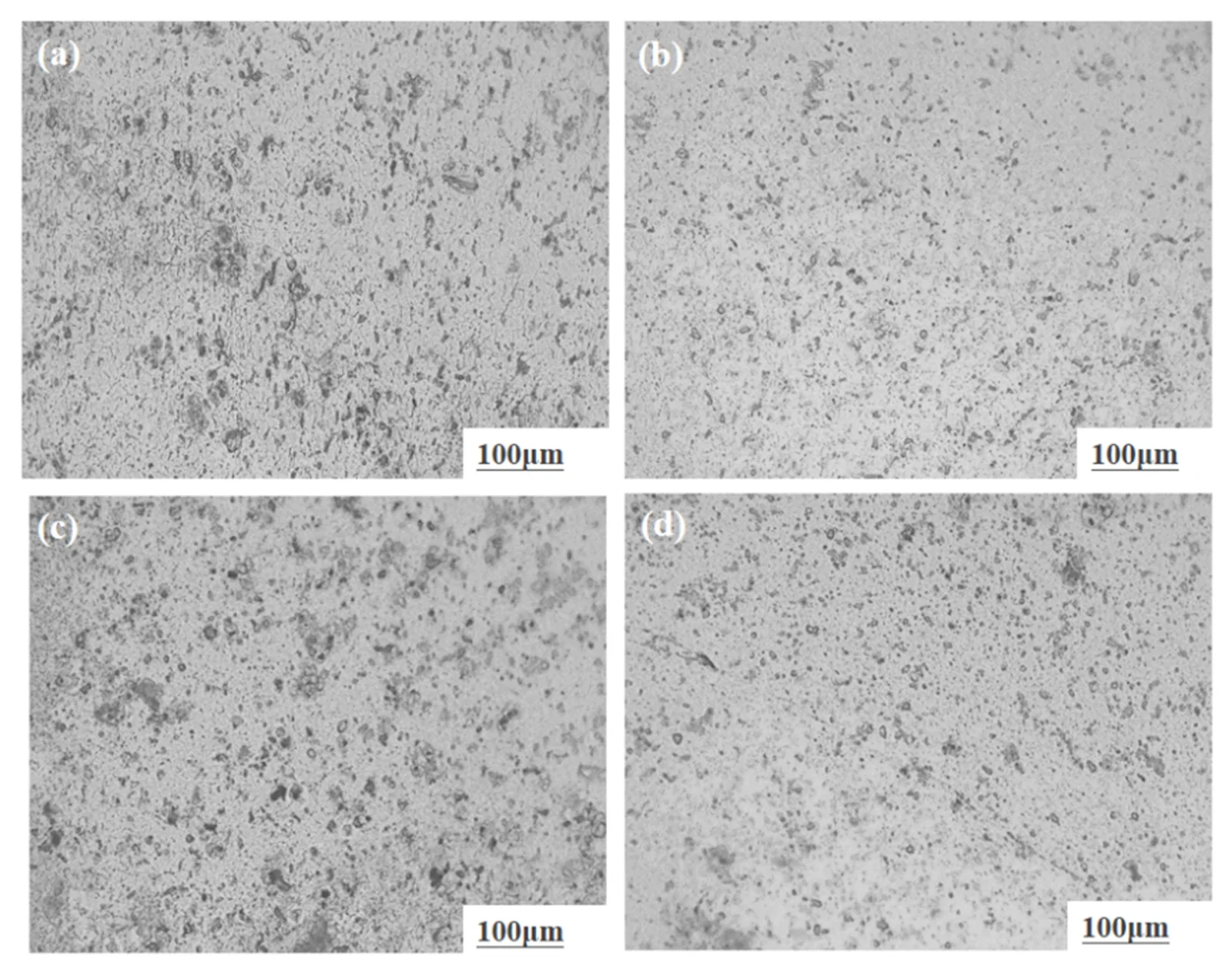
Grain morphology in the TMAZ region of FSSW-repaired joints under different repair speeds: (**a**) 800 r/min; (**b**) 1200 r/min; (**c**) 1600 r/min; and (**d**) 2000 r/min.

To further analyze the microstructure morphology of FSSW, Figure 7 presents the grain morphology in the TMAZ zone of the FSSW-repaired joints of 2024 aluminum alloy under different repair rotational speeds. When the rotational speed gradually increases from 800 r/min to 2000 r/min, the heat generated by the high-speed rotation of the stirring head increases during the hole repair process. As the heat input gradually increases, the material in the repair zone is gradually plasticized. Through the viscosity between the materials, it gradually drives the material in the TMAZ zone to flow. The grains in the TMAZ zone undergo mechanical stirring and heat input from the stirring head, resulting in obvious plastic deformation. When the repair speed is 800 r/min, the heat input is relatively small, and the degree of plastic deformation of the grains is relatively low, as shown in Figure 7a. As the repair speed increases to 1200 r/min and 1600 r/min, the heat input during the spot welding process increases, and the degree of plastic deformation of the grains intensifies, as shown in Figure 7b,c. When the repair speed is 2000 r/min, the grain arrangement in the TMAZ zone is finer and more compact, as shown in Figure 7d.

**Figure 8:**
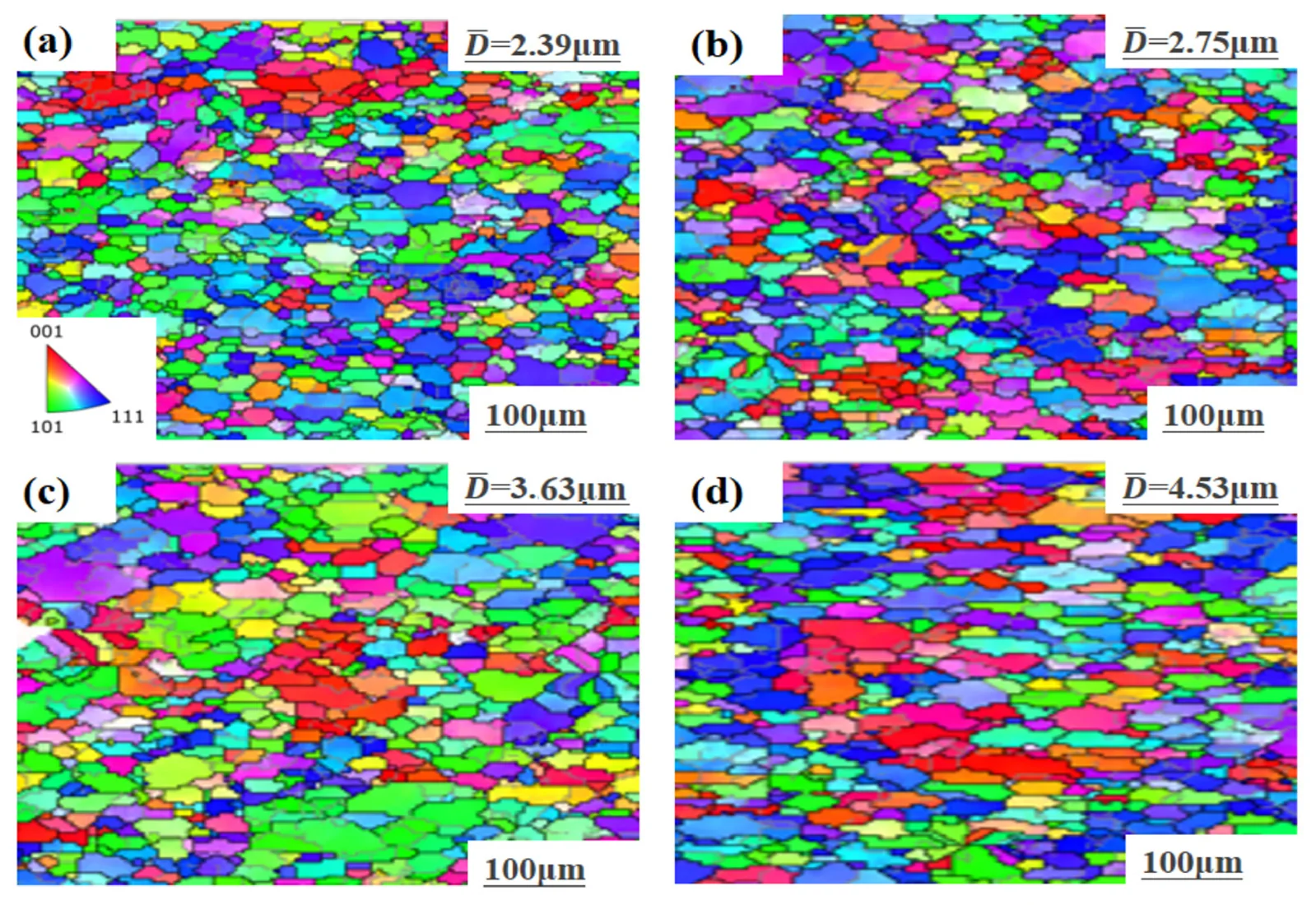
Grain morphology in the NZ region of FSSW-repaired joints under different repair speeds: (**a**) 800 r/min; (**b**) 1200 r/min; (**c**) 1600 r/min; and (**d**) 2000 r/min.

Figure 8 shows the grain size morphology of the NZ zone in the FSSW-repaired joint of 2024 aluminum alloy with hole defects at repair rotational speeds of 800 r/min to 2000 r/min. During the FSSW repair process, the NZ zone undergoes recovery and recrystallization under the coupling effect of thermal cycling and plastic deformation, resulting in grain refinement and the formation of a uniform microstructure. Meanwhile, the grain size in the NZ zone of the FSSW-repaired joint increases with the increase in the repair rotational speed. When the repair speed is 800 r/min, the average grain size of the NZ zone of the FSSW-repaired joint is 2.39 μm, as shown in Figure 8a. When the repair rotational speed is 1200 r/min, the average grain size of the NZ zone of the FSSW-repaired joint is 2.75 μm, as shown in Figure 8b. When the repair rotational speed is 2000 r/min, the average grain size of the NZ zone of the FSSW-repaired joint is 4.53 μm. Compared with the repair speed of 800 r/min, the grain size of the NZ zone of the repaired joint increases by 89.5%. This is mainly due to the fact that as the rotational speed increases from 800 r/min to 2000 r/min, the NZ zone of the FSSW-repaired joint experiences continuous accumulation of thermal input and, under the strong thermomechanical stirring cycle, undergoes microstructure evolution dominated by dynamic recrystallization (DRX), accompanied by grain growth at high temperatures [22].

**Figure 9:**
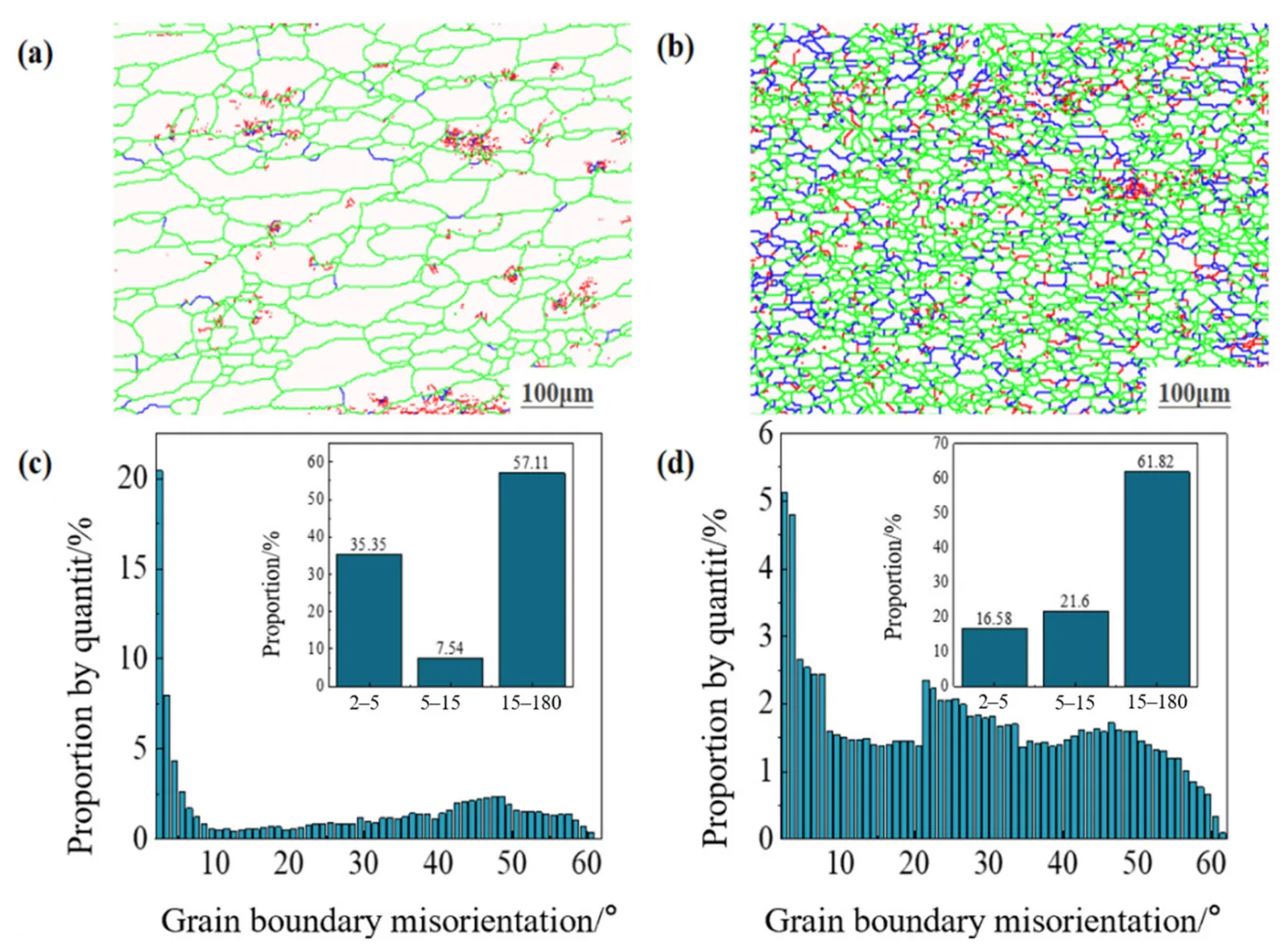
Grain boundaries and misorientation distributions of the repaired joint at 800 r/min: (**a**) BM region grain boundary diagram; (**b**) NZ region grain boundary diagram; (**c**) the misorientation of the BM region; and (**d**) the misorientation of the NZ region.

Figure 9 shows the grain boundary and orientation difference distribution of the FSSW-repaired joint under the repair welding speed of 20 mm/min and the repair rotational speed of 800 r/min. The average grain size of the base metal zone is approximately 55 μm, and the proportion of HAGBs ranging from 15° to 180° is 57.11%. From the BM zone to the NZ zone, with the increase in thermal input and mechanical stirring effect, dynamic recrystallization occurs in the FSSW-repaired joint, forming a fine equiaxed crystal structure, which leads to a significant increase in the number of grain boundaries in the NZ zone. The proportion of large-angle grain boundaries ranging from 15° to 180° is 61.82%, which increases by 8.25% compared to the BM region. In addition, with a large amount of heat input, the DRX in the NZ zone is enhanced accordingly. Many HAGB grains are gathered around the large-sized grains in Figure 9a. In the BM zone of the FSSW-repaired joint, the proportion of 2–5° low angle grain boundaries (LAGBs) are 35.35%, and that of 5–15° is 7.54%, as illustrated in Figure 9c. However, the proportion of LAGBs in the NZ area of the FSSW-repaired joint with angles ranging from 2 to 5° decreases from 35.35% to 16.58%. The proportion of those with angles ranging from 5 to 15° increases from 7.54% to 21.6%, as shown in Figure 9d. This is primarily because during the FSSW repair process, when the grains of LAGBs are subjected to external forces, they can generate deformation dislocations. Due to the high orientation difference within the grains, an increasing number of grains with HAGBs are formed. Meanwhile, the grains of HAGBs are subjected to mechanical stirring by the stirring head, forming recrystallized grains. Therefore, under the repair speed of 800 r/min, the proportion of HAGB grains in the NZ zone significantly increases, and the proportion of DRX also increases substantially.

**Figure 10:**
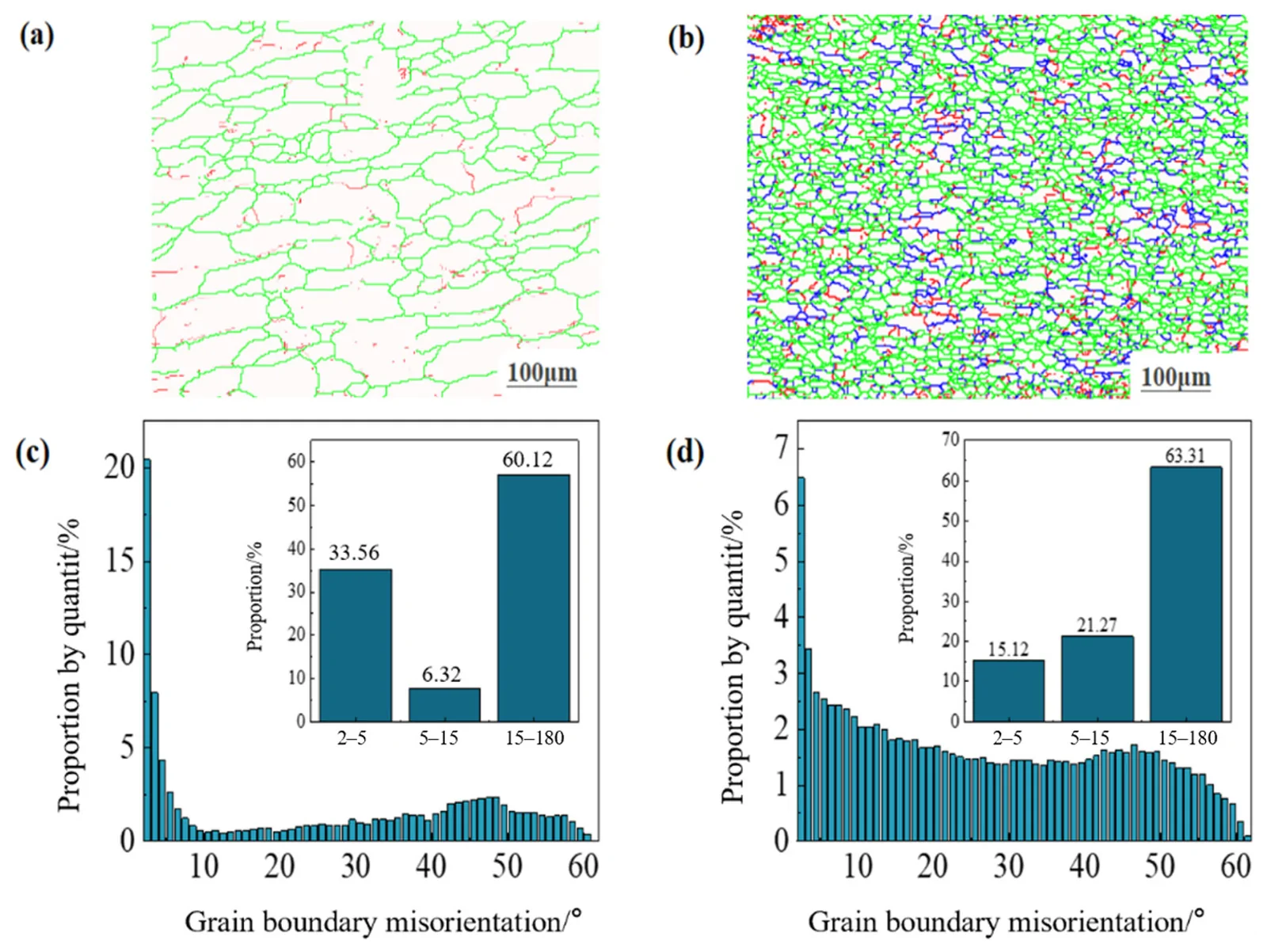
Grain boundaries and misorientation distributions of the repaired joint at 1200 r/min: (**a**) BM region grain boundary diagram; (**b**) NZ region grain boundary diagram; (**c**) the misorientation of the BM region; and (**d**) the misorientation of the NZ region.

Figure 10 shows the grain boundary and orientation difference distribution of the FSSW-repaired joint under the repair welding speed of 20 mm/min and the repair rotational speed of 1200 r/min. As can be seen in Figure 10a, around the larger-sized grains in the BM region, there are many small grains clustered together. The proportion of LAGBs ranging from 2° to 5° is 33.56%, and the proportion of HAGBs ranging from 15° to 180° is 60.12%, as shown in Figure 10c. From the BM zone to the NZ zone, with the further intensification of heat input and mechanical stirring, the number of grain boundaries in the NZ zone increases significantly. The proportion of HAGBs ranging from 15° to 180° is 63.31%, which increases by 10.85% compared to the BM zone in Figure 10b. Under the influence of substantial heat input, DRX in the NZ zone is accordingly enhanced, as shown in Figure 10d. As can be seen in the comparison between Figure 10c,d, the proportion of LAGBs ranging from 2° to 5° decreased from 33.56% to 15.12%. This is mainly because during the repair process, the grains of LAGBs absorb a large amount of heat input and mechanical stirring, resulting in deformation dislocations. The proportion of HAGB grains increased, and the DRX proportion of grains in the NZ zone increased significantly.

**Figure 11:**
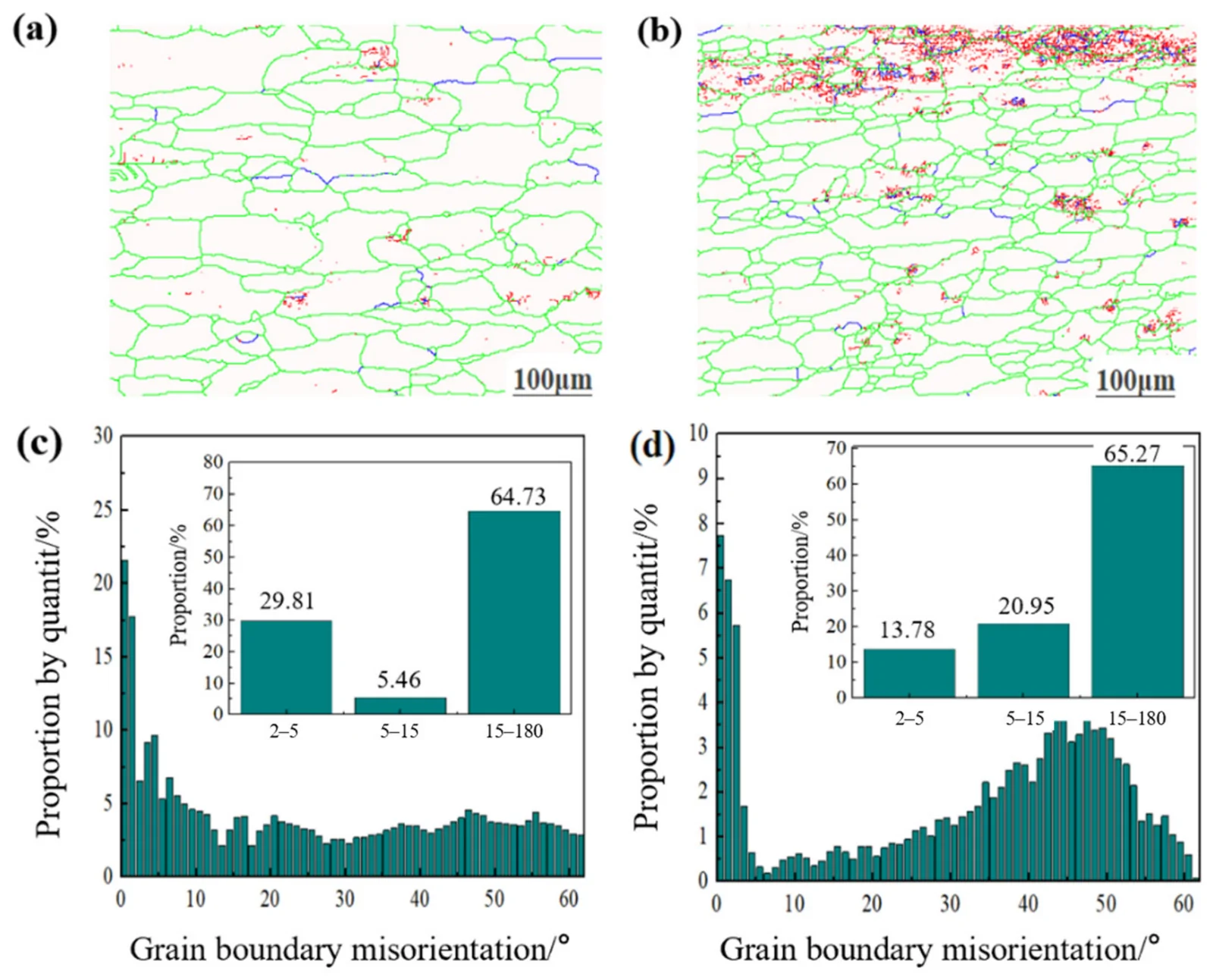
Grain boundaries and misorientation distributions of joints at 2000 r/min: (**a**) BM region grain boundary diagram; (**b**) NZ region grain boundary diagram; (**c**) the misorientation of the BM region; and (**d**) the misorientation of the NZ region.

Figure 11 shows the grain boundary and orientation difference distribution of the FSSW-repaired joint when the repair welding speed is 20 mm/min and the repair rotational speed is 2000 r/min. As the repair rotation speed continuously increases, the degree of deformation experienced by the repaired joint structure gradually intensifies. Particularly, both the BM zone and the NZ zone undergo severe plastic deformation of the grains, resulting in a significant degree of recovery and recrystallization. Compared with the results of repairing at 800 r/min and 1200 r/min (Figure 9 and Figure 10), the orientation angle distribution results in Figure 11c,d show that the content of LAGBs is significantly reduced, while the proportion of HAGBs is notably increased. Among them, the proportions of LAGBs within the range of 2–5° in the BM and NZ regions are 29.81% and 13.78%, respectively; the proportions of HAGBs within the range of 15–180° are 64.73% and 65.27%, respectively. Compared with the grain boundaries in the BM region shown in Figure 11a, the number of grain boundaries in the NZ region significantly increased. Meanwhile, during the FSSW repair process, the stir head generates a large amount of heat input and continuous plastic deformation in the NZ zone, causing recrystallization of the grains in the NZ zone and the formation of subgrain structures within the crystals, as shown in the red area in Figure 11b.

**Figure 12:**
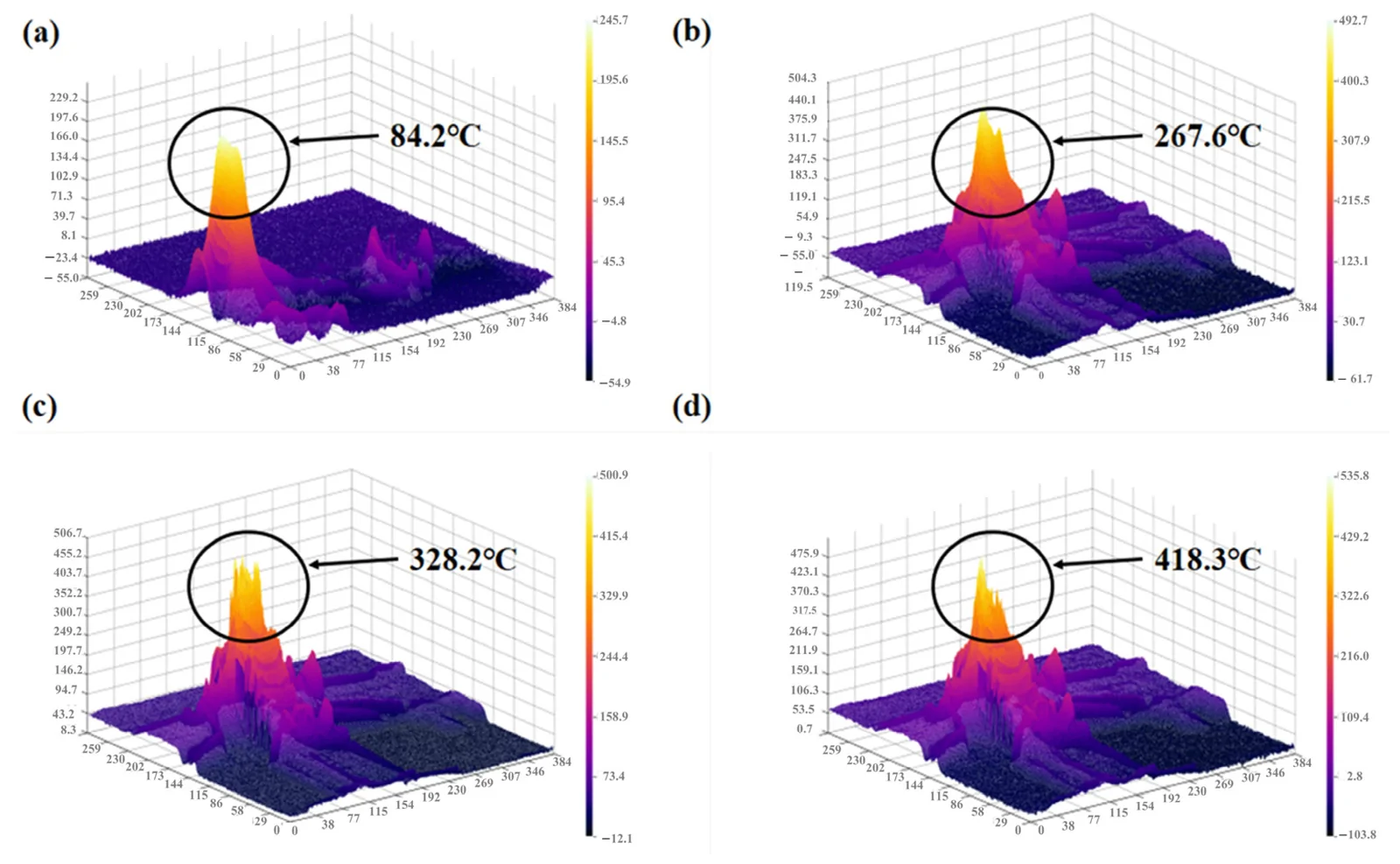
Three-dimensional infrared thermal imaging diagram of the FSSW-repaired joint: (**a**) 800 r/min; (**b**) 1200 r/min; (**c**) 1600 r/min; and (**d**) 2000 r/min.

Figure 12 presents the three-dimensional infrared thermal imaging diagrams of FSSW-repaired joints under the repair welding speed of 20 mm/min and different repair rotational speeds ranging from 800 r/min to 2000 r/min. The temperature distribution during the point contact repair process was analyzed using the AnalyzIR 1.16.100.8109 infrared thermal imaging analysis software in combination with different repair process parameters. The AnalyzIR infrared thermal imaging temperature map indicates that the temperature distribution is generally non-uniform, with the highest temperature observed in the shoulder region. From the center to the edge of the stirring head, the temperature exhibits a gradient variation. When the repair rotation speeds are set at 800 r/min and 1200 r/min, the corresponding maximum temperatures reaches 84.2 °C and 267.6 °C, as shown in Figure 12a,b, respectively. As the repair speed increases continuously, the frictional heat generated by the FSSW-repaired joint rises significantly, and the overall temperature as shown by infrared thermal imaging increases. When the rotational speeds are 1600 r/min and 2000 r/min, the maximum temperature of the FSSW-repaired joint reaches 328.2 °C and 418.3 °C, respectively. Meanwhile, the width between the peaks of the three-dimensional thermal imaging temperature map increases, as shown in Figure 12c,d.

**Figure 13:**
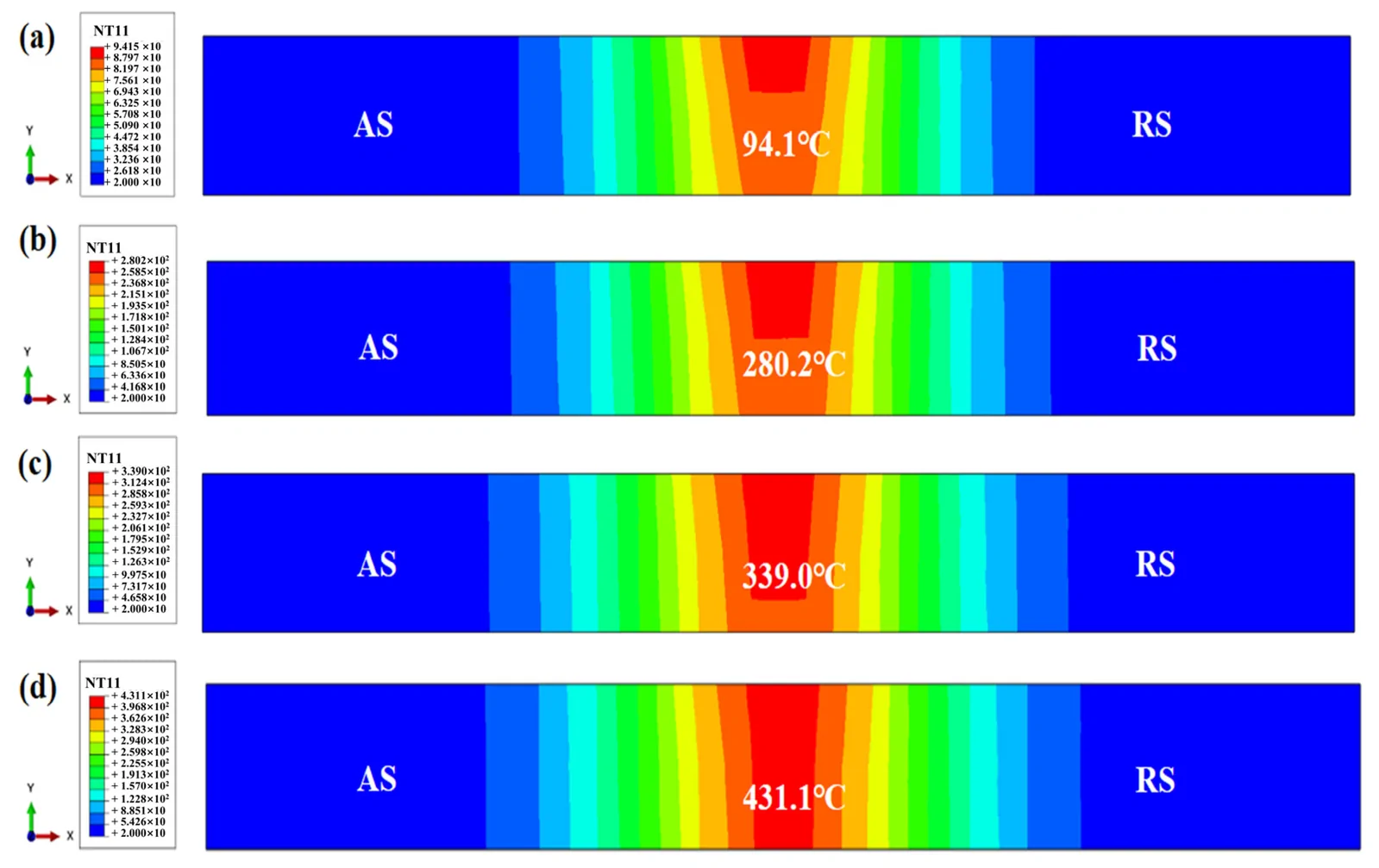
Cross-sectional temperature distribution contour of the XY plane of the FSSW-repaired joint at the same time: (**a**) 800 r/min; (**b**) 1200 r/min; (**c**) 1600 r/min; and (**d**) 2000 r/min.

Figure 13 shows the cross-sectional temperature distribution cloud map in the XY plane at the same time for FSSW-repaired joints under different repair speeds of 800 r/min–2000 r/min. The temperature distribution shows a symmetrical trend on both sides of the repair centerline. In the temperature distribution cloud map, due to the main heat input coming from the intense friction between the stirring head and the base material, the temperature distribution cloud map appears red. As the distance from the repair center increases, the temperature decreases, and the temperature distribution cloud map appears blue. When the repair speed is 800 r/min, the highest temperature is 94.1 °C, and the area affected by the heat input is very small, as shown in Figure 13a. When the repair speed is 1200 r/min in Figure 13b, the maximum temperature of the FSSW-repaired joint is 280.2 °C. When the repair speed increases to 1600 r/min and 2000 r/min, the maximum temperatures are 339.0 °C and 431.1 °C, respectively, and the width and depth of the heat input affected area increase significantly, as shown in Figure 13c,d. The temperature field simulation results indicate that sufficient heat input has fully plasticized the material around the void, effectively promoting uniform thermomechanical mixing between the tool and the void defect, thereby achieving more consistent material flow continuity. Meanwhile, the NZ region undergoes significant thermal cycles and plastic deformation during welding, providing the thermomechanical driving conditions for dynamic grain evolution. Furthermore, in the ABAQUS simulation results, when the repair speeds are 1600 r/min and 2000 r/min, the temperature distribution of the FSSW-repaired joint only differs by 3% from the AnalyzIR thermal imaging temperature shown in Figure 12. Therefore, the simulation results are consistent with experimental measurements, indicating a high degree of accuracy in the thermomechanical coupling model for FSSW repair.

**Figure 14:**
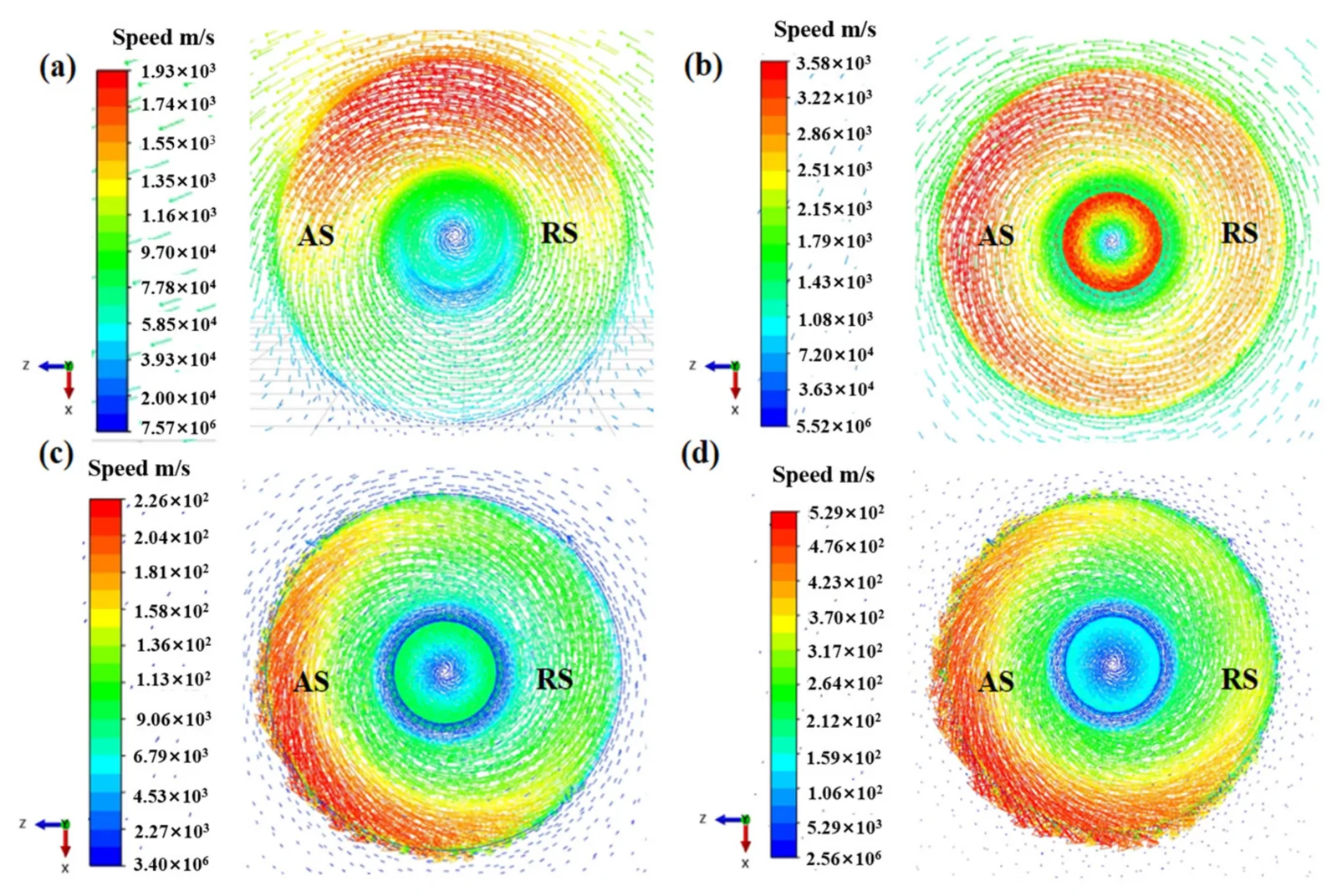
XZ plane velocity distribution of FSSW-repaired joints at the same time: (**a**) 800 r/min; (**b**) 1200 r/min; (**c**) 1600 r/min; and (**d**) 2000 r/min.

Figure 14 shows the velocity distribution in the XZ plane of the FSSW-repaired joint at the same moment. Driven by the stirring head, the repair material rotates and flows symmetrically relative to the center of the cavity defect. Different velocity gradients form a velocity difference, which drives the material towards the cavity defect. During the repair process at a rotational speed of 800 r/min, the insufficient rotational speed results in inadequate material flow and backfilling of the repair material, as shown in Figure 14a. The repair material fails to achieve proper integration with the surrounding base material, which is consistent with the cross-sectional morphology in Figure 5a.

At 800 r/min, the maximum flow velocity is only 1.93 × 10^−3^ m/s, which is insufficient to drive the material to fully fill the cavity; when the rotational speed increases to 2000 r/min, the maximum flow velocity reaches 5.29 × 10^−2^ m/s, which is approximately 27 times the value at 800 r/min. In terms of velocity gradient, the velocity difference between the advancing side (AS) and the retreating side (RS) grows gradually with the rise in rotational speed. At 2000 r/min, the velocity difference between the AS and RS reaches 3.7 × 10^−2^ m/s. The velocity gradient generates a shear effect inside the material, which promotes the mixing of plastic materials, breaks the interface between the consumable stirring pin and the base material, and facilitates the formation of metallurgical bonding. A repaired joint with excellent repair performance is obtained, as shown in Figure 5d.

The filling ratio is defined as the proportion of the filled volume to the original volume of the prefabricated hole, which is calculated based on cross-sectional metallographic photographs. At 800 r/min, due to insufficient material fluidity, large-area voids exist in the repair zone, and the filling ratio is only approximately 62%. With the increase in rotational speed, the filling ratio improves continuously: it reaches about 85% at 1200 r/min and 95% at 1600 r/min. When the rotational speed reaches 2000 r/min, the hole defects are eliminated and the filling ratio attains 100%, achieving high-quality repair of the hole defects. The above quantitative indicators fully support the conclusion that increasing the rotational speed can enhance material flow and improve the defect filling effect. The relevant quantitative analysis is presented in Section 3, Results and Discussion. Furthermore, the simulation results in Figure 14 are in agreement with the metallographic observations of the FSSW-repaired joint in Figure 5. Both demonstrate that a higher repair rotation speed leads to a better repair quality of the FSSW joint.

**Figure 15:**
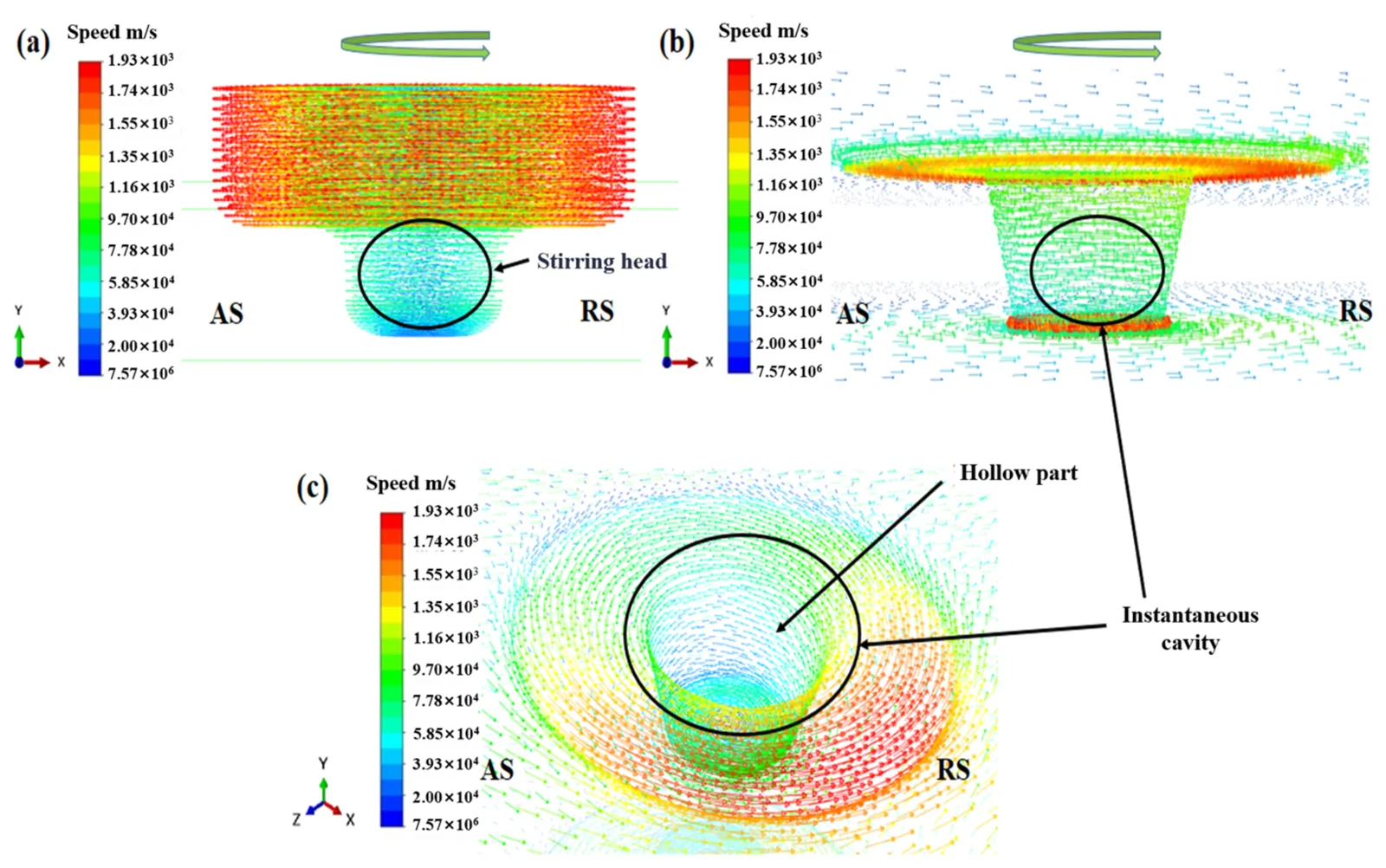
Velocity vector of FSSW-repaired joint at 1600 r/min: (**a**) near the stir-welding head; (**b**) distribution of repair material flow near the hole; and (**c**) around the hole.

During the FSSW repair process, the flow behavior of the repair material has a decisive influence on the formation of the repaired joint. Figure 15 shows the transverse cross-sectional velocity vector maps of the repaired joint at a repair rotational speed of 1600 r/min. Driven by the stirring tool; the repair material flows rapidly around the tool. During the plunging stage, material flows into the cavity from the periphery and the bottom of the stirring tool, while at the same time, the material in the hollow region is thrown outward toward the surroundings of the tool under the action of centrifugal force, forming an outward flow. At this point, a transient cavity is formed in the center of the hollow region, as shown in Figure 15b,c. Under the action of the stirring tool, the surrounding material continuously flows into this cavity, establishing a filling flow circulation until the entire hole defect region is filled.

**Figure 16:**
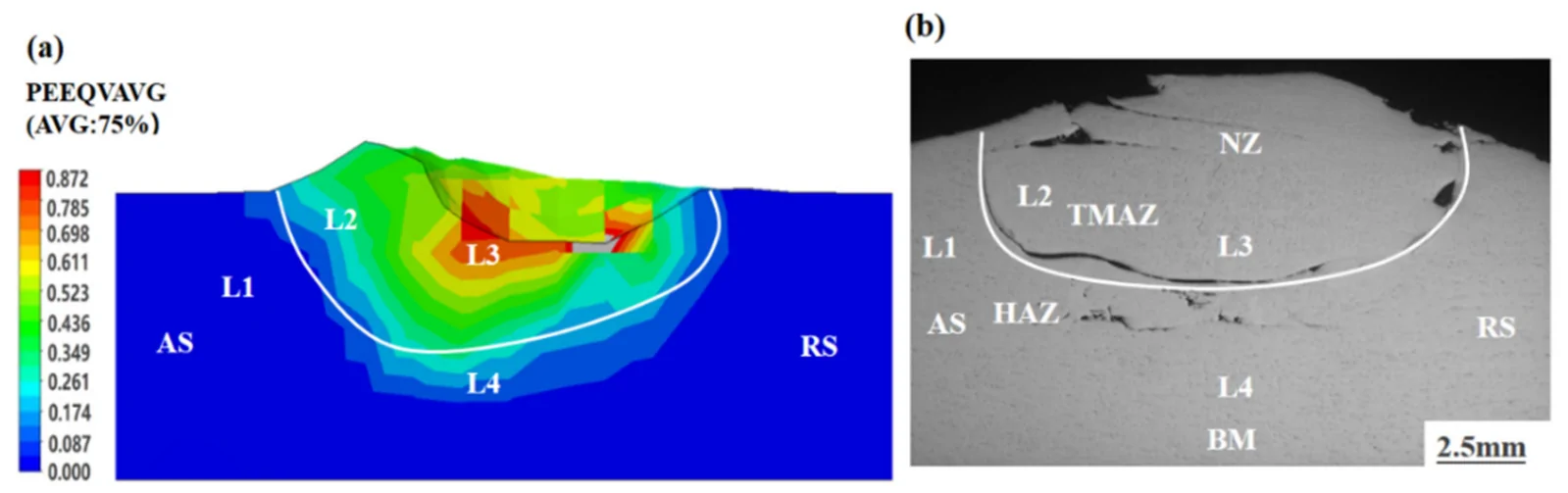
The equivalent strain cloud diagram and the microstructure morphology of the FSSW-repaired joint at a rotational speed of 800 r/min: (**a**) ABAQUS simulation equivalent effect variable cloud map; (**b**) FSSW-repaired joint metallographic morphology.

Figure 16 presents the equivalent plastic strain nephogram and the metallographic morphology of the repaired joint cross-section under a repair welding speed of 20 mm/min and a repair rotation speed of 800 r/min. The correspondence between the equivalent plastic strain results and the metallographic observation results is emphasized. During the FSSW repair process, the material near the bottom and around the periphery of the stirring head experiences a relatively high equivalent strain, as shown in Figure 16a. The NZ zone and the TMAZ zone experience relatively high plastic deformation during the FSSW repair process. Particularly, the equivalent strain in the NZ zone (L3 area) is higher than that in the TMAZ zone (L1 area). However, the equivalent strain is low near the top of the stirring head and the lower surface of the repair material. The equivalent strain in the HAZ zone (L4 area) on the lower surface of the repair material remains almost zero, indicating that almost no plastic deformation has occurred in this area. Figure 16b shows the metallographic structure morphology of the FSSW-repaired joint. It can be observed that plastic deformability is the strongest in NZ. The microstructure of this region is also the most compact. Many pores are generated at the boundary between the TMAZ and the HAZ. The material from the stirring tool does not completely merge with the surrounding material, and some relatively large voids are present. Overall, the equivalent strain contour from the ABAQUS simulation shows a reasonable correspondence with the main microstructural zones of the FSSW-repaired joint in Figure 16b, although local discrepancies at the zone boundaries remain evident. This further validates the rationality of the thermal–mechanical coupling model used for the FSSW repair process.

**Figure 17:**
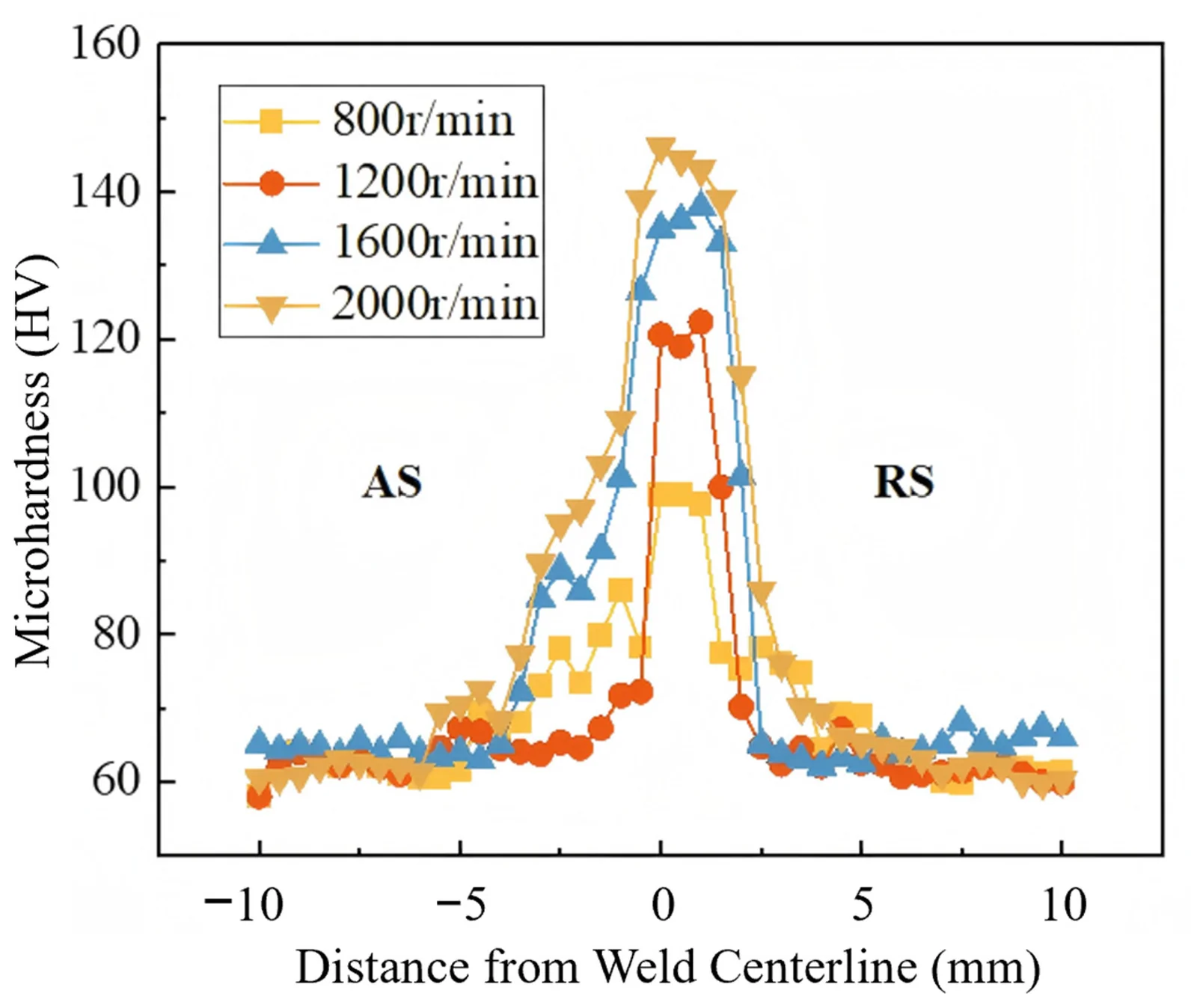
Effect of different repair rotation speeds on the microhardness distribution of FSSW-repaired joints.

Figure 17 shows the microhardness distribution of FSSW-repaired joints under different repair rotational speeds. The microhardness values of the FSSW-repaired joints exhibit significant differences in different regions, and the hardness distribution presents an N-shaped pattern, with high hardness in the center and low hardness on both sides. Among them, the hardness of NZ is significantly higher than that of the BM and TMAZ zones. The average hardness of NZ is approximately 120 HV, while that of the BM is approximately 65 HV. Under repair rotation speeds of 800 r/min and 1200 r/min, the hardness values from the HAZ to the NZ increase from 70 HV and 65 HV to 100 HV and 120 HV, respectively. Under repair rotation speeds of 1600 r/min and 2000 r/min, the hardness values from the HAZ to the NZ increase from 65 HV and 69 HV to 135 HV and 146 HV, respectively. Meanwhile, as the repair rotation speed increases, the width of the weld NZ gradually increases, and the farther the repair material on both sides of the NZ is from the weld, the lower its hardness value. This is mainly attributed to the increase in heat input as the repair rotation speed rises from 800 r/min to 2000 r/min. The higher heat input expands the softening range of the material around the welding zone, thereby promoting plastic flow over a wider region and leading to significant hardening in the welding zone during the repair welding process.

**Figure 18:**
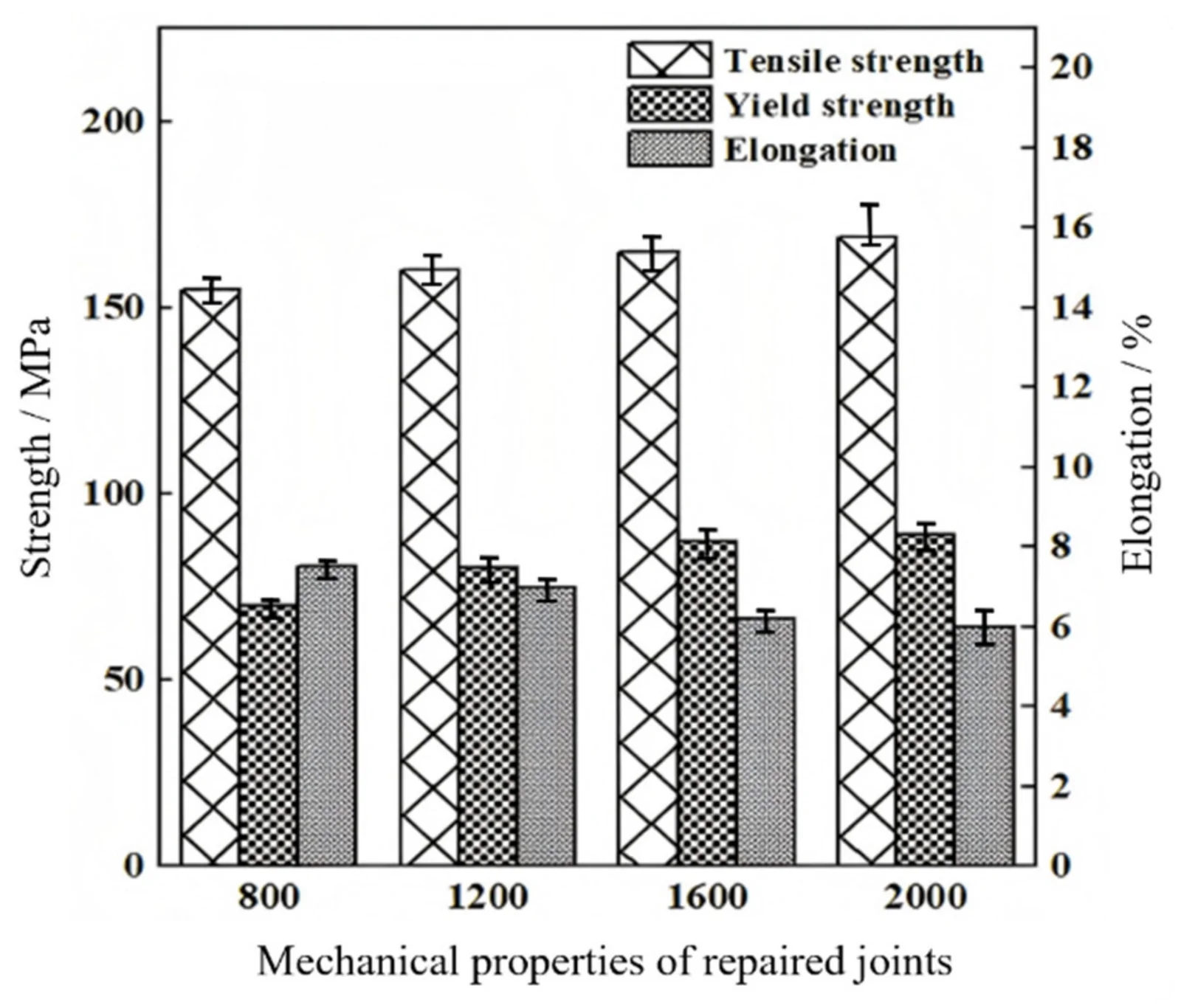
Mechanical properties of FSSW-repaired joints under different repair speeds.

Figure 18 shows the mechanical properties of FSSW-repaired joints under different repair rotational speeds. According to Figure 18, as the repair rotation speed increases from 800 r/min to 2000 r/min, both the tensile strength and yield strength of the FSSW-repaired joint continuously increase, although the increase in yield strength is more gradual. When the repair rotation speed reaches 2000 r/min, both the tensile strength and yield strength of the repaired joint achieves their maximum values of 169 MPa and 89 MPa, respectively. Compared with the values at a repair rotation speed of 800 r/min, the tensile strength and yield strength of the FSSW-repaired joint increase by 9% and 27%, respectively. However, the elongation of the FSSW-repaired joint gradually decreases with increasing repair rotation speed. The elongation is measured at 7.5% with a repair speed of 800 r/min, 7% for 1200 r/min, and 6% for 2000 r/min. This is primarily because as the repair rotation speed increases, the frictional heat generated by the high-speed rotation of the stirring tool plasticizes the base material. Consequently, stress and strain concentrations occur at the weakest area of the FSSW-repaired joint, which disrupts the uniformity of deformation and leads to a decrease in the elongation of the joint. The results indicate that the FSSW-repaired joint exhibits good strength.

**Figure 19:**
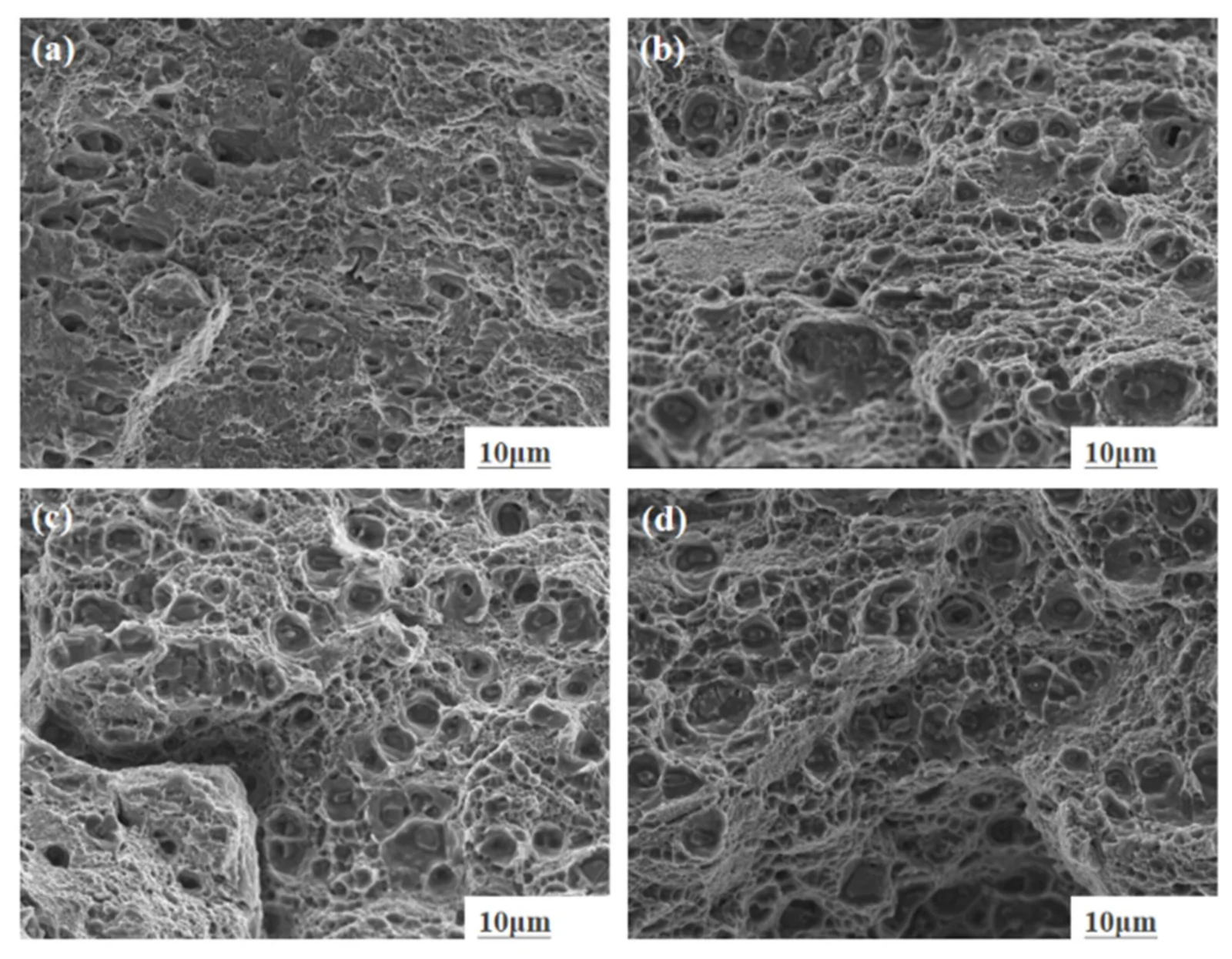
Morphology of tensile fractures of FSSW-repaired joints under different repair speeds: (**a**) 800 r/min; (**b**) 1200 r/min; (**c**) 1600 r/min; and (**d**) 2000 r/min.

Figure 19 presents the fracture morphologies of the 2024 aluminum alloy FSSW-repaired joint obtained at repair rotation speeds ranging from 800 to 2000 r/min. Fracture of the FSSW-repaired joint occurs under axial tensile loading, with the fracture surface located in the Nugget Zone (NZ) of the repaired area. The fracture surface is oriented at a 45-degree angle to the loading direction. As can be seen in the figure, the fracture mode of the FSSW-repaired joint is predominantly ductile fracture. The fracture surface exhibits a characteristic dimple pattern with a mixture of large and small dimples of varying sizes. At a repair rotation speed of 800 r/min, the 2024 aluminum alloy FSSW joint exhibits relatively low plasticity. As shown in Figure 19a, the fracture surface is characterized by numerous shallow dimples with a relatively low population density, accompanied by the presence of minor tear ridges surrounding the dimpled regions. At a repair rotation speed of 1200 r/min, the fracture surface of the FSSW-repaired joint exhibits an increased number of dimples of varying sizes, along with the presence of more tear ridges, as shown in Figure 19b. At a repair rotation speed of 1600 r/min, the fracture surface of the FSSW-repaired joint contains many small-sized dimples, as shown in Figure 19c. At a repair rotation speed of 2000 r/min, the fracture surface of the FSSW-repaired joint exhibits a significantly increased number of small-sized dimples with greater depth and a more uniform distribution.

## 4. Conclusions

In this study, the FSSW repair process is employed to address pre-existing hole defects in 2024 aluminum alloy thin-walled components. The evolution of microstructure and mechanical properties of the FSSW-repaired joints is investigated under different repair rotational speeds, and the main conclusions are as follows:

(1) When the repair rotation speed is 800 r/min, void defects exist in the NZ zone of the repair center. As the repair rotational speed increases, the macroscopic surface quality of the joints, repaired by FSSW for the 2024 aluminum alloy hole defects, shows significant improvement. When the rotational speed reaches 2000 rpm, the surface of the repaired zone is smooth and flat, and the bottom of the repaired zone achieves complete metallurgical bonding with the base metal (BM), with no observed welding defects such as porosity or cracks.
(2) Under the thermomechanical action of the welding tool, the Nugget Zone (NZ) of the FSSW-repaired joint undergoes significant grain refinement strengthening. The grains in this region are refined through dynamic recrystallization, transforming into a uniform equiaxed structure with the formation of subgrain structures within the grains. When the rotational speed increases from 800 to 2000 r/min, the grain size in the Nugget Zone (NZ) of the FSSW-repaired joint increases from 2.39 μm to 4.53 μm.
(3) The temperature distribution contour obtained from the ABAQUS simulation shows good agreement with that from the AnalyzIR thermal imaging test, effectively validating the temperature field of the FSSW repair process. The temperature increases as one approaches the center of the repair zone. The velocity contour plot from the simulation reveals that increasing the repair tool rotation speed significantly enhances the material flow behavior. At a rotational speed of 2000 r/min, the surrounding material is strongly driven to fully fill the hollow region, forming a filling flow circulation.
(4) The mechanical properties of the FSSW-repaired joint improve with increasing repair rotation speed. When the rotation speed reaches 2000 r/min, the peak values of tensile strength and yield strength are 180 MPa and 90 MPa, respectively. Compared with the 2024-O aluminum alloy base material, the tensile strength and yield strength increase by 6% and 20%, respectively. The fracture mode of the repaired joint is ductile fracture, with all fractures located within the Nugget Zone (NZ) of the repaired region. As the repair rotation speed increases, the number of small dimples on the joint’s surface significantly increases.

## Figures and Tables

**Table 1:** Chemical composition and mechanical properties of 2024-O aluminum alloy.

Chemical Composition (%)						Mechanical Properties		
Cu	Mg	Si	Mn	Fe	Al	Tensile strength (MPa)	Yield strength (MPa)	Elongation (%)
4.2	1.53	0.47	0.66	0.53	Bal.	170	75	12

**Table 2:** Johnson–Cook constitutive model parameters.

A (MPa)	B (MPa)	C	n	m	T_ref_ (°C)	T_melt_ (°C)	ε_0_
270	154.3	0.001	0.22	0.8	24	585	1

## Data Availability

The original contributions presented in this study are included in the article. Further inquiries can be directed to the corresponding author.
